# Antimicrobial Applications of Green Synthesized Bimetallic Nanoparticles from *Ocimum basilicum*

**DOI:** 10.3390/pharmaceutics14112457

**Published:** 2022-11-14

**Authors:** Pragati Rajendra More, Carla Zannella, Veronica Folliero, Francesco Foglia, Romualdo Troisi, Alessandro Vergara, Gianluigi Franci, Anna De Filippis, Massimiliano Galdiero

**Affiliations:** 1 Department of Experimental Medicine, Section of Microbiology and Clinical Microbiology, University of Campania “L. Vanvitelli”, Via De Crecchio, 7, 80138 Naples, Italy; 2 Department of Chemical Sciences, University of Napoli Federico II, Via Cinthia, 80126 Napoli, Italy; 3 Department of Medicine, Surgery and Dentistry, “Scuola Medica Salernitana”, University of Salerno, 84081 Baronissi, Italy

**Keywords:** bimetallic nanoparticles, *Ocimum basilicum*, phytoconstituents, antibiotic resistance, antimicrobial agents

## Abstract

Antibiotic resistance is an important and emerging alarm for public health that requires development of new potential antibacterial strategies. In recent years, nanoscale materials have emerged as an alternative way to fight pathogens. Many researchers have shown great interest in nanoparticles (NPs) using noble metals, such as silver, gold, and platinum, even though numerous nanomaterials have shown toxicity. To overcome the problem of toxicity, nanotechnology merged with green chemistry to synthesize nature-friendly nanoparticles from plants. Here, we describe the synthesis of NPs using silver (AgNPs) and platinum (PtNPs) alone or in combination (AgPtNPs) in the presence of *Ocimum basilicum* (*O. basilicum*) leaf extract. *O. basilicum* is a well-known medicinal plant with antibacterial compounds. A preliminary chemical–physical characterization of the extract was conducted. The size, shape and elemental analysis were carried out using UV–Visible spectroscopy, dynamic light scattering (DLS), and zeta potential. Transmission electron microscopy (TEM) confirmed polydisperse NPs with spherical shape. The size of the particles was approximately 59 nm, confirmed by DLS analysis, and the polydisperse index was 0.159. Fourier transform infrared (FTIR) demonstrated an effective and selective capping of the phytoconstituents on the NPs. The cytotoxic activities of AgNPs, PtNPs and AgPtNPs were assessed on different epithelial cell models, using the 3-[4.5-dimethylthiazol-2-yl]-2.5-diphenyltetrazolium bromide (MTT) cell proliferation assay, and discovered low toxicity, with a cell viability of 80%. The antibacterial potential of the NPs was evaluated against *Escherichia coli* (*E. coli*), *Enterococcus faecalis* (*E. faecalis*), *Klebsiella pneumonia* (*K. pneumoniae*), and *Staphylococcus aureus* (*S. aureus*) strains. Minimum inhibitory concentration (MIC) assays showed AgPtNP activity till the least concentration of NPs (3.15–1.56 µg/mL) against ATCC, MS, and MDR *E. coli*, *E. faecalis*, and *S. aureus* and the Kirby–Bauer method showed that AgPtNPs gave a zone of inhibition for Gram-positive and Gram-negative bacteria in a range of 9–25 mm. In addition, we obtained AgPtNP synergistic activity in combination with vancomycin or ampicillin antibiotics. Taken together, these results indicate that bimetallic nanoparticles, synthesized from *O. basilicum* leaf extract, could represent a natural, ecofriendly, cheap, and safe method to produce alternative antibacterial strategies with low cytotoxicity.

## 1. Introduction

The rapid spread of microbial epidemics with resistant strains of microbes is becoming an alarming cause of morbidity and mortality in the human health domain. Among the multiple mechanisms by which bacteria develop resistance to antibiotics, the production of enzymes that can alter their activity, and changes in the efflux pathways, that restrict the passage of drugs, represent the most common [1]. Reports published by the World Health Organization (WHO) have shown that multidrug-resistant (MDR) bacterial infections are a huge health threat all over the world [2]. Padilla-Cruz et al. suggested that if the trend continues in the same direction with the same speed, it will lead to 10 million people dying every year by 2050 [1]. The WHO has identified some bacteria as high-risk pathogens, with intense focus on the ESKAPE pathogens (*Enterococcus faecium*, *Staphylococcus aureus*, *Klebsiella pneumonia*, *Acinetobacter baumannii*, *Pseudomonas aeruginosa*, *Enterobacter* spp.) playing an important role in this global health concern [2]. To slow down the spread of antibiotic resistance, the design of novel antibacterial agents is necessary.

Nanotechnologies, which aim to control molecular and three-dimensional structures to create molecularly precise nanomaterials and nanodevices, cooperate with life and medical sciences in a promising way. Noble metal nanoparticles have great potential to be used as antimicrobial agents, such as in medical devices, wastewater treatment, packaging, and dentistry [3,4]. Metal precursors can be converted into metal NPs, with typical sizes from 1 to 100 nm. In detail, NPs with smaller sizes have a large surface/volume ratio and a strong interaction with bacteria, exerting targeted and prolonged antimicrobial activity, even at smaller dosages [5]. It is well known that metal NPs have wide-ranging antimicrobial effects against several microorganisms, including Gram-positive and Gram-negative bacteria and also viruses [6]. The physical and chemical methods of synthesizing NPs have limitations concerning the stabilization of synthesized NPs, and also have environmental hazard risks. For this reason, green synthesis of metal NPs is preferred, in which phytochemicals with antioxidant or reducing properties are used as a potential precursor for synthesizing NPs and reducing metal ions.

In this study, we used a leaf extract of *O. basilicum* as a reducing agent. The leaves of *O. basilicum* have a high range of secondary metabolites, like phenolic compounds, and polyphenols, such as flavonoids and anthocyanin [7,8]. They also have extensive diversity in *Ocimum* oils [9,10]. The presence of various biomolecules makes this a good candidate to be used as the reducing agent in the process of synthesizing NPs. In recent years, various studies have reported that NPs designed with noble metals, such as silver (Ag), gold (Au), and platinum (Pt), exhibited strong and sustainable antibacterial action [1,11]. In addition, AgNPs have been used for biosensing in different pharmaceutical industries, biomedical imaging, and drug delivery and also for designing antimicrobial surfaces, cosmetics paints, and plastics [8,9].

In recent years, bimetallic NPs have attracted the attention of researchers for their unique optical, electronic, magnetic, and catalytic properties, which are significantly distinguishable from their monometallic counterparts. Bimetallic NPs are formed by the combination of two different types of metallic NPs, which, acting synergistically, can have a variety of morphologies and structures [12]. A green synthesis of metal NP is based on the use of plants, bacteria, and fungi extracts. Indeed, phytochemicals with antioxidant or reducing properties are used as potential precursors for synthesizing NPs by reducing the metal ions. According to different reports it has been suggested that metal ions show strong potential to reduce faster than others with weak reduction ability. On this basis, different bimetallic nanoparticle syntheses have been carried out. Some examples are silver–gold, gold–platinum, gold–palladium [13], palladium–platinum [14], platinum–copper [15], and nickel–copper [15] nanoparticles with potential antioxidant, anticoagulant, and thrombolytic activities. *O. basilicum* is designated as within the group of medicinal and aromatic plants, with various pharmaceutical applications [16,17]. In addition, we used silver and platinum as noble metals to synthesize these NPs. The antimicrobial potential of silver has been proven since ancient times [18,19].

This study aimed to synthesize silver–platinum bimetallic nanoparticles (AgPtNPs) in the presence of *O. basilicum* leaf extract as a biological reducing agent. We assessed the antimicrobial applications of the synthesized NPs, and, in addition, we tested a combination of this extract and noble metal to assess the synergistic effect against different bacteria [16,17].

## 2. Materials and Methods

### 2.1. Materials

Silver nitrate (AgNO_3_, ≥99%), potassium terachloroplatinate (II) (K_2_PtCl_4_ ≥ 99.9%), 2,2 diphenyl-1-picrylhydrazyl (DPPH), Mueller Hinton agar, and Mueller Hinton broth, were acquired from Sigma-Aldrich (St. Louis, MO, USA). All components used for cell culture, such as 3-(4, 5-dimethylthiazol-2-yl)-2, 5-diphenyltetrazolium bromide (MTT), Dulbecco’s Modified Eagle’s Medium (DMEM), fetal bovine serum (FBS), penicillin streptomycin solution, were acquired from Microtech srl (Naples, Italy). All solutions were prepared in distilled water and the leaves of *O. basilicum* were collected from farmers in Naples, Italy.

### 2.2. Tested Microorganisms

*Escherichia coli* (*E. coli*, ATCC 11229), Enterococcus faecalis (*E. faecalis*, ATCC 29212), *Klebsiella pneumoniae* (*K. pneumoniae*, ATCC 10031), and *Staphylococcus aureus* (*S. aureus*, ATCC 6538) strains were obtained from American Type Culture Collection (ATCC, Manassas, VA, USA). The multi-drug resistant (MDR) bacteria and multi-sensitive (MS) bacteria were collected from clinical isolates from the Unit of Virology and Microbiology of University Hospital “Luigi Vanvitelli”, Naples, Italy. Bacterial identification was carried out via MALDI-TOF MS (Bruker Daltonics, Bremen, Germany), and the antibiotic resistance patterns were evaluated by the BD Phoenix system (Becton Dickinson, Franklin Lakes, NJ, USA). Resistance profiles for ATCC and clinical isolate tested strains (Table 1 and Table 2) are reported in our previous work [20].

### 2.3. Preparation of Plant Extract and Synthesis of Bimetallic Nanoparticles

To extract the phytochemicals or essential oils from the leaves of *O. basilicum*, we followed the extraction procedure described by Saba et al. [8]. Fresh leaves of *O. basilicum* were collected from a local farmer in Naples, Italy. Then, the fresh leaves were washed thoroughly with double distilled water until the dust was completely removed from the leaves. After washing, the leaves were crushed to release the phytochemicals from the leaves. These crushed leaves were placed in a 500 mL Erlenmeyer flask containing 100 mL distilled water. This flask was heated at 80 °C for 40 min with constant shaking. Once the solution had cooled, it was filtered through Whatman filter paper No. 1. The filtrate was collected and stored at 4 °C for further use in the synthesis of the NPs.

**Figure 1:**
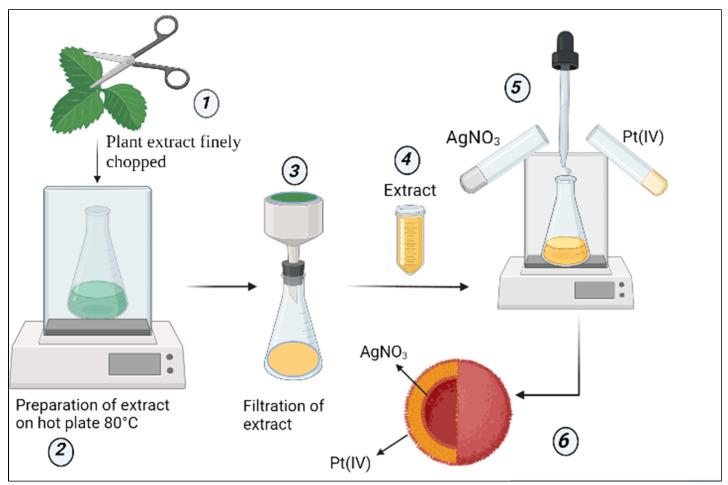
Schematic representation of synthesis of AgPtNPs from *O. basilicum*. (1) Fresh leaves of *O. basilicum* were chopped finely (2) Then, the finely chopped leaves were used for the preparation of the exact on a hot plate at 80 °C (3) The prepared extract was then filtered through a Whatman filter paper No.1 (4) This extract was used for the preparation of the bimetallic NPs (5) At 80 °C, and under continuous shaking conditions in the presence of two noble metals, namely Silver nitrate and Potassium tetrachloroplatinate (II), synthesis of the NPs was carried out (6). The hypothesis was that the NPs formed would possess an inner core of silver and outer cover of platinum.

Figure 1 illustrates the workflow to explain the synthesis process of NPs.

### 2.4. Biosynthesis of NPs

To synthesize AgPtNPs, 10 mL of leaf *O. basilicum* extract was added to a flask containing 50 mL of distilled water. The flask was shaken continuously at 600 rpm at 80 °C. Then 1 mM of AgNO_3_ solution was added dropwise, followed by dropwise addition of 1 mM of K_2_PtCl_4_. The reaction was performed at 80 °C under constant shaking for 60 min. After 30–40 min reaction, the color changed from colorless to dark yellowish green. At the 60 min endpoint, the reaction mixture was stored in dark conditions for the next 24 h. After 24 h, this reaction mixture was centrifuged and washed several times with H_2_O and centrifuged to remove any untreated salts and extracts to obtain the dry pellet. This pellet was then kept at 40 °C to acquire the NPs in powdered form. The same synthesis protocol was followed to prepare monometallic NPs (AgNPs and PtNPs). The reaction was monitored with UV-Vis spectroscopy to observe the surface plasmon resonance from the NPs’ surfaces. The NPs of desired weight dispersed in distilled water, and the NPs and distilled water ratio was kept at 1:1 [21,22,23]. Further, sonification for 10 min enabled further characterization and biological applications [24,25]

### 2.5. Dynamic Light Scattering and Zeta Potential

Particle stability was checked by Dynamic light scattering (DLS) and surface charges were measured by zeta potential via Zetasizer Nano S (Malvern PANalytical, Malvern, UK).

### 2.6. Fourier Transformed Infrared Spectroscopy

Dried powder samples of purified nanoparticles were diluted to prepare KBr pellets (1% in weight, 1 mm optical path) to be used for FTIR spectroscopy in a transmission mode. Vibrational spectra of the extract-reduced AgNPs and AgPtNPs were recorded by an FTIR spectrometer (model Nicolet 5700) in the range of 400–4000 cm^−1^ [26].

### 2.7. Transmission Electron Microscopy (TEM) Analysis

The NPs’ morphologies and sizes were confirmed by dropping a dispersed solution onto a carbon-coated copper grid and then allowing this to dry, before performing analysis using TEM at 200 kV voltage. The analysis was carried out by using a FEI Tecnai G2, while the images were captured by FEI TEM (Version 4.7 SP3).

### 2.8. Disk Diffusion Test

A Kirby–Bauer disk diffusion susceptibility test was used to screen the antibacterial activity of AgPtNPs against different ATCC bacteria, and MDR and MS clinical isolates. Briefly, fresh colonies were used to prepare an inoculum at 0.5 McFarland turbidity. The bacterial suspension was homogeneously plated on Muller Hinton agar plates (Oxoid, Basingstoke, Hampshire, MA, USA). Different paper disks were placed on the agar plate, and loaded with 10 µL of AgPtNPs, AgNPs and PtNPs. A Vancomycin disk (Thermo Fisher Scientific, Waltham, MA, USA) was used for Gram positive bacteria and an ampicillin disk for Gram negative bacteria (Thermo Fisher Scientific, Waltham, MA, USA). Finally, plates were incubated at 37 °C for 24 h until the zone of inhibition was observed.

### 2.9. Minimum Inhibition Concentration (MIC) Assay

A MIC value was calculated by the microdilution method in 96 well plates [20]. Each sample was serially diluted in a range from 0.78 to 100 µg/mL, while ampicillin and vancomycin were used as positive controls. In addition, untreated bacteria were used as negative control. In brief, 1 × 10^6^ Unit Forming Colony (CFU)/mL of bacterial suspension was adjusted and 50 µL of inoculum was added to each well to have the final concentration of 5 × 10^5^ CFU/well. Lastly, the plate was incubated at 37 °C for 24 h. After 24 h of incubation the plate was checked with a microtiter plate reader (Sunrise, Tecan Austria GmbH, Austria) at 600 nm. The lowest concentration showing 100% of bacterial growth inhibition was considered as the MIC value for respective bacterial strains.

### 2.10. Time Killing Test

To better understand the NPs’ kinetics of action against bacteria, the time killing test was performed in accordance with the American Society for Testing and Materials International (ASTM) standard guidelines and following the protocol described by Loo et al. [27]. The OD value of the bacterial inoculum was adjusted to 10^6^ CFU/mL of bacteria, which were added to the MHB, and NPs were added to obtain the final concentration of 2xMIC, MIC, and ½ MIC, in a final volume of 1 mL/tube. Each mixture was incubated at 37 °C under orbital shaking (180 rpm) and, then, 100 µL aliquot of suspension was serially diluted and plated on Mueller Hinton Agar after different incubation periods of 0, 2, 4 and 24 h. The plates were incubated at 37 °C viable CFU and a time-kill curve was plotted using each time period. The time-kill curve was compared to the positive and negative control curves.

### 2.11. Checkerboard Assay

To determine the MIC values of antibiotics and NPs in combination, two-fold dilutions were performed in 96 well plates. The interaction between the antibiotics and NPs was determined by fractional inhibitory concentration (FIC), for which antibiotics were indicated as FIC_Ab_ and NPs as FIC_NP_ [28]. The FIC index was calculated by using the following formula:

FIC_AB_ = MIC of antibiotic + MIC of NP combination/MIC of antibiotics alone

FIC_NP_ = MIC of antibiotic + MIC of NP combination/MIC of nanoparticles

FIC index combination = FIC_Ab_ + FIC_NP_

A synergistic effect of the antibiotics and NPs was indicated when the FIC index value was ≤0.5, while they were considered antagonistic if the FIC value was ≥4, and additive if the FIC value was in the range 0.5–1, while it is indifferent 1 and 4.

### 2.12. MTT Assay

To assess the cytotoxicity of NPs, a 3-(4,5-dimethylthiazol-2-yl)-2,5-diphenyltetrazolium bromide (MTT) assay was performed on the African green monkey cell line (Vero), Human immortalized keratinocytes cells (HaCaT) and Colon carcinoma cells (Caco-2) cell line. Cells were plated in DMEM (Thermo Fisher Scientific, Waltham, MA, USA), supplemented with 1% penicillin–streptomycin and 10% fetal bovine serum (Thermo Fisher Scientific, Waltham, MA, USA), at 37 °C, with 5% CO_2_ in a humid environment. A density of 2 × 10^4^ cells/well was seeded into 96-well plates and incubated for 24 h. The day after, cells were treated with AgPtNPs, AgNPs and PtNPs at different concentrations ranging from 3.15 to 100 μg/mL. After 24 h, MTT solution was added to each well and plates were incubated for an additional 3 h at 37 °C. Then, formazan crystals were dissolved with DMSO 100% and the absorbance was measured at OD 570 nm using a microplate reader (Tecan, Männedorf, Switzerland).

### 2.13. Statistical Analysis

Data are expressed as mean ± standard deviation (SD) of three independent experiments. One-way analysis of variance (ANOVA) and post-hoc Dunnett’s test were performed by using the Prism 6.0 software (Graph Pad, San Diego, CA, USA). Differences were considered significant at a *p*-value < 0.05.

## 3. Results

### 3.1. Synthesis of NPs and Characterization

**Figure 2:**
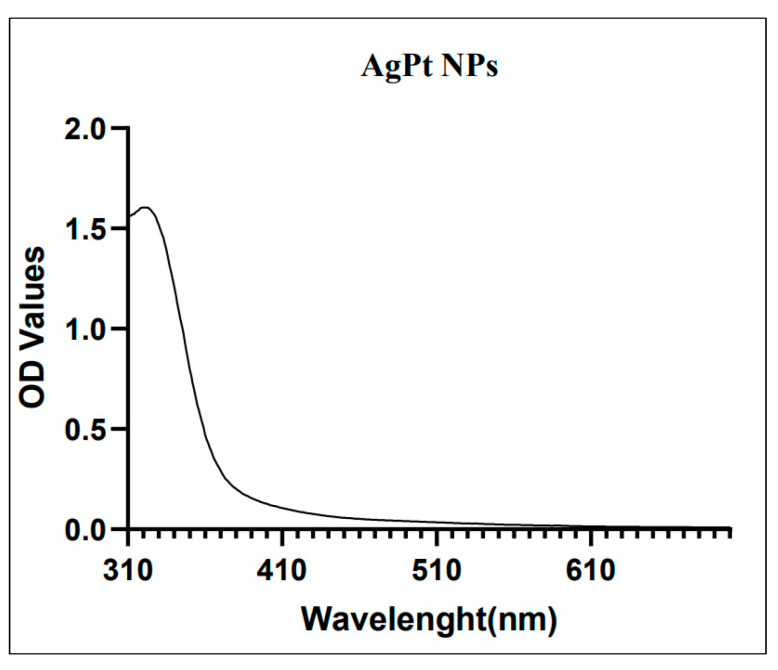
UV–Vis spectral wavelength for a batch of synthesized AgPtNPs (peak at 323 nm).

To evaluate the reduction of noble metal we monitored the color change in the reaction. Initially, the color of *O. basilicum* was yellow. After the addition of AgNO_3_ and K_2_PtCl_4_ and under constant shaking at 80 °C, the color change of the reaction was observed as transforming from dark yellow to a yellowish green, indicating the formation of AgPtNPs. After 1 h of reaction, UV-Vis spectra of *O. basilicum* capped AgPtNPs exhibited the peak around 320–450 nm (Figure 2).

**Figure 3:**
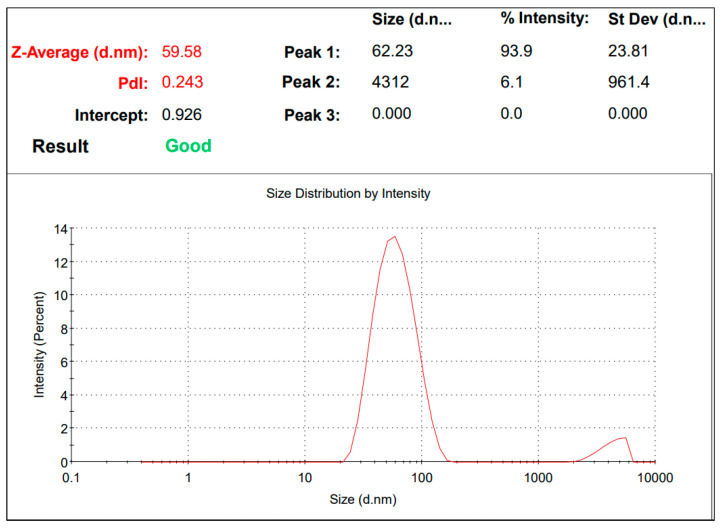
DLS analysis. The size of synthesized NPs is 59 nm.

Further, AgPtNPs’ size and charge were measured via DLS and Zeta potential. The AgPtNP showed negative charge zeta potential, maybe due to strong adsorption of phytochemicals on the formed NPs. Likewise, they improved their stability and prevented an aggregation of particles. The size of particles was approximately 59 nm, as confirmed by DLS analysis, and the polydisperse index was 0.159 (Figure 3). The low degree of PDI indicated good quality and polydisperse particles. The above parameters made these NPs ideal for their biological activities.

**Figure 4:**
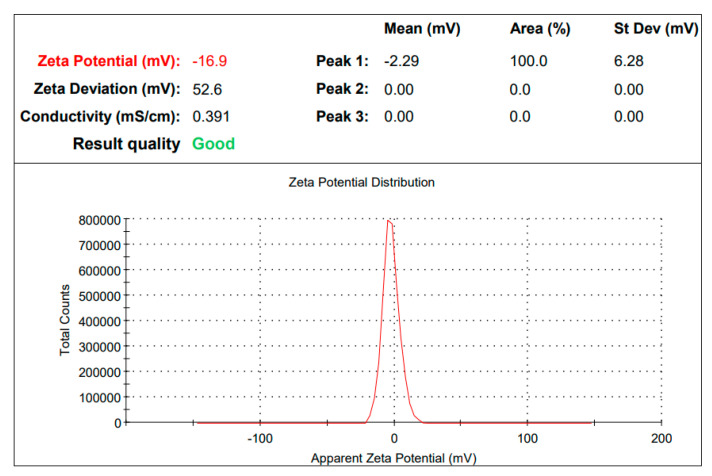
Zeta potential value for AgPtNP is −16.9.

Generally, the zeta potential values of NPs should be within the range of +30 mV to −30 mV to be considered as stable. For AgPtNP the zeta value was observed to be −16 mV (Figure 4).

**Figure 5:**
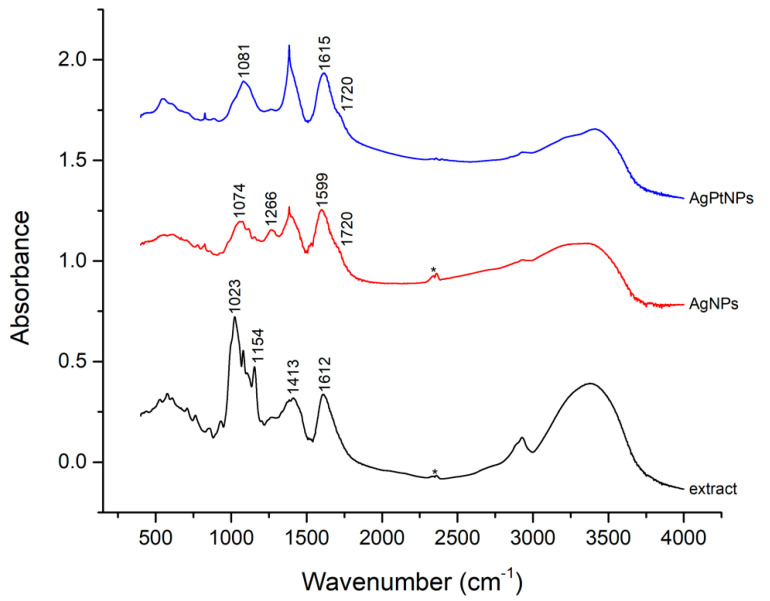
FT−IR spectra of basil extract (without NPs), AgNPs extract, and AgPtNPs extract. The star refers to carbon dioxide.

FTIR spectra were recorded for the extracts, AgNPs and AgPtNPs (Figure 5), along with metallic precursors used as control. A comparative analysis of the above FTIR spectra indicated a simplified spectrum, suggesting a selective capping of *O. basilicum* extract components covering the metal NPs. The comparison of missing and additional bands between metal NP extracts and natural extract spectra could give some preliminary clues on the coating mechanism. Particularly, AgNP and AgPtNP extracts spectra did not exhibit the intense IR band at 1023 cm^−1^ (consistent with C-C or C-O stretching) dominating the natural extract, and they showed a much lower CH stretching envelope (in the range 2800–2900 cm^−1^) and OH/NH stretching around 3300–3400 cm^−1^. This evidence suggested a minor variability in organic content in the NP extract. The disappearing major features at 1023 cm^−1^ could not be definitively assigned, though they might have been due to the remotion, during NP capping, of a basil essential oil [29] or of a polysaccharide content [30]. Moreover, AgNP and AgPtNP spectra showed the appearance of a shoulder at 1720 cm^−1^, suggesting a possible involvement of carbonyl groups in the NP coating mechanism. Finally, when comparing the bimetallic AgPtNPs and monometallic AgNPs (Figure 5), they looked quite similar, also showing a mild amount of AgNO_3_ precursor still being present (1384 and 823 cm^−1^).

**Figure 6:**
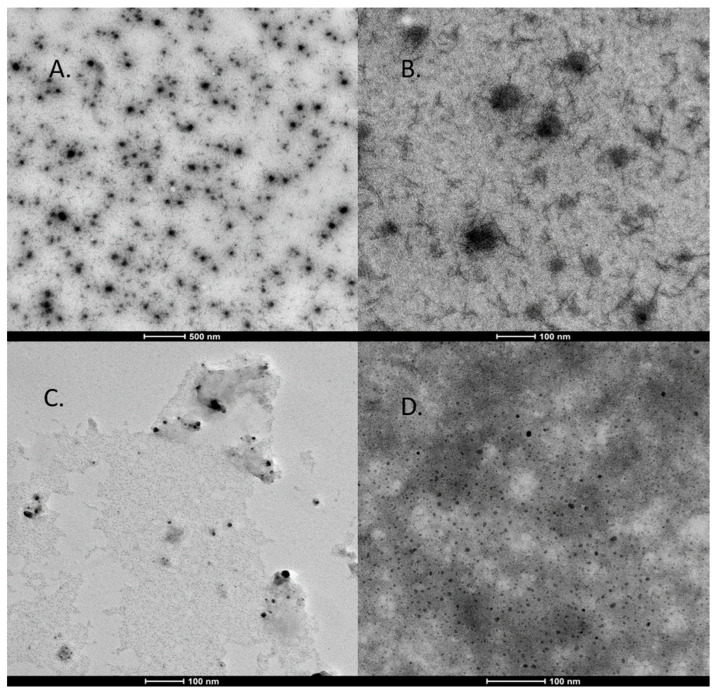
TEM analysis of bimetallic NPs. (**A**,**B**): Bimetallic nanoparticles with the internal core composed by Ag and the outer coating consisting of Pt. (**C**) Very tiny silver NPs (**D**) Platinum hollow NPs.

Further, the morphological characterizations of the AgPtNPs were confirmed by TEM analysis. Very small independent particles forming nanoclusters with an overall size distribution from 20 to 80 nm and an average of 59 nm were identified (Figure 6). Interestingly, the AgPtNPs showed an inner core of silver and outer core of platinum (Figure 6 A,B). While Figure 6C only shows AgNPs with very tiny sizes around 5–10 nm, Figure 6D shows the hollow PtNPs with average sizes of 10–20 nm. The results suggested that the AgPtNPs had a spherical shape with a shell structure, in which the inner core was of silver and the outer core of platinum. The chemical mechanism of the structure formation is not known yet, and needs to be explored in further studies.

### 3.2. Evaluation of Antibacterial Activity by Disk Diffusion Test

The disc diffusion assay was performed to get a preliminary idea about the antimicrobial activity of the AgPtNPs. The assay was performed in comparison with monometallic NPs (AgNPs and PtNPs). The antimicrobial activity was analyzed against different bacterial strains obtained by ATCC, MS, and MDR *E. coli*, *E. faecalis*, *K. pneumoniae*, and *S. aureus*. We observed a significant antimicrobial effect in the zone of inhibition against all bacterial pathogens. AgPtNPs showed a zone of inhibition for Gram-positive and Gram-negative bacterial strains ranging from 9–25 mm. While the AgNPs showed an inhibition zone of 9–10 mm, the PtNPs showed inhibition zones in a range of 6–9 mm (Table 3).

### 3.3. Evaluation of Antibacterial Activity by MIC

**Figure 7:**
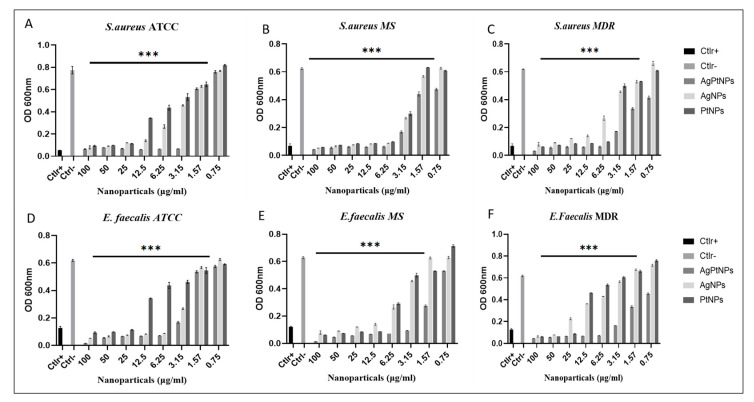
Analysis of AgPtNPs by MIC against ATCC, MS and MDR Gram-positive. (**A**–**C**) indicate the analysis against all *S. aureus* strains and (**D**–**F**) against *E. faecalis.* Ctlr+ indicates bacteria treated with antibiotic, while Ctlr− represents non-treated bacteria. Data are relative to Ctlr – and represent the mean ± standard deviation (SD). Significant diff. among means (*** *p* < 0.05).

**Figure 8:**
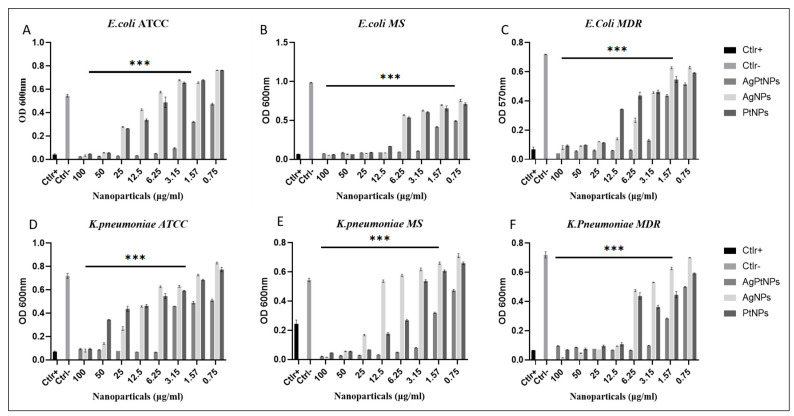
Analysis of AgPtNPs by MIC against ATCC, MS and MDR Gram-negative bacterial pathogens. (**A**–**C**) indicate the analysis against all *E. coli* and (**D**–**F**) against *K. pneumoniae*. Data are means of three independent experiments. Ctlr+ indicates bacteria treated with antibiotic, while Ctlr− represents not-treated bacteria. Data are relative to Ctlr – and represent the mean ± standard deviation (SD). Significant diff. among means (*** *p* < 0.05).

Further, to understand the minimum inhibitory concentration, we performed a microdilution assay by means of the broth method. The AgPtNPs showed good activity till the least concentration of NPs (3.15–1.56 µg/mL) and in the same study conducted with AgNPs and PtNPs, the AgNPs showed MIC in the range of 25–3.15 µg/mL, and for PtNPs it was 12.5–3.15 µg/mL. AgPtNPs MIC was studied for different bacterial strains. Gram-positive and Gram-negative bacteria, such as ATCC, MS, and MDR *K. pneumoniae*, *E. coli*, *E. faecalis*, and *S. aureus*, were used for this assay. The concentration of AgPtNPs was 0.75 to 100 µg/mL, while the same concentrations were used for monometallic NPs (AgNPs and PtNPs). The bacterial suspension of 1 × 10^6^ CFU/mL was placed in a 96 well plate, and to each well was added 50 µL of inoculum. After 24 h of incubation at 37 °C in the 96-well plates, turbidity was observed at different concentrations. The lowest concentration of NPs showing no turbidity was considered as the MIC value. The AgPtNPs showed good activity till the least concentration of NPs (3.15–1.56 µg/mL) against ATCC, MS, and MDR *E. coli*, *E. faecalis*, and *S. aureus* and the AgPtNPs showed a significant rise in susceptibility at an MIC value of 3.15 µg/mL. When the same study was conducted with AgNPs and PtNPs, the AgNPs showed MIC in the range of 25–3.15 µg/mL, and for PtNPs it was 12.5–3.15 µg/mL. The exact mechanism of action of AgPtNPs was not yet clear. We hypothesized that they could exert a direct action on the bacterial membrane, destabilizing it and forming pores that altered its integrity. Figure 7 shows the data for the Gram-positive pathogens and Figure 8 for Gram negative, while the Table 4 and Table 5 indicate the MIC values for *S. aureus* and *E. faecalis* and *E. coli* and *K. pneumoniae* ATCC, MS, and the MDR strains, respectively.

### 3.4. Evaluation of Synergistic Effect of AgPtNPs

**Figure 9:**
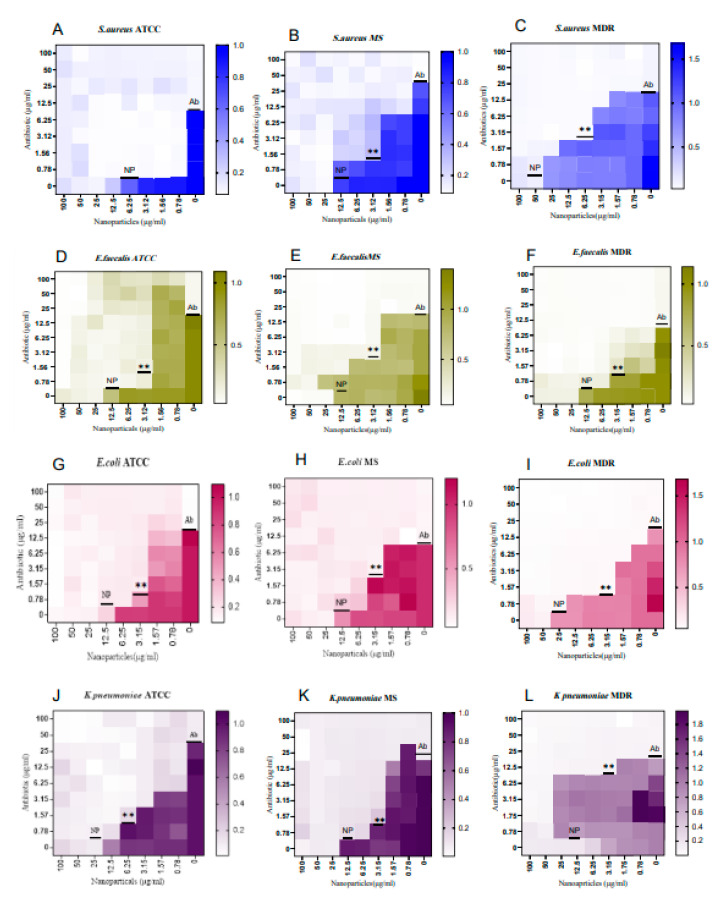
Synergistic, additive or antagonistic effect against AgPtNPs and antibiotics (ampicillin and vancomycin). (**A**–**C**) panels clarify the synergistic effect against *S. aureus* ATCC, MS and MDR bacterial strain. (**D**−F) panels indicate the additive effect against *E. faecalis* ATCC, MS and MDR bacterial strains. (**G**–**I**) panels indicate the synergistic effect against *E. coli* ATCC, MS and MDR bacterial strains. Lastly, (**J**–**L**) panels indicate the additive and synergistic effects against *K. pneumoniae* ATCC, MS and MDR bacterial strain. NP: nanoparticles; Ab: antibiotic. Data are means of three independent experiments. Significant diff. among means **: *p* < 0.01.

After evaluating the antimicrobial efficacy of AgPtNPs by MIC at different concentrations, an antibiotics checkerboard assay was performed. To analyze the synergistic activity of AgPtNPs with Ampicillin and Vancomycin, the FIC index was calculated for both NPs and antibiotics. If the resulting values were within the range of 0.5–4, the effect was called additive or indifferent. Synergistic activity was indicated when the resulting FIC index was ≤0.5, while antagonism was indicated when the FIC index was ≥4 and above. In this study, after determining the FIC index of NPs and antibiotics, we tested this mixture against different ATCC, MS, and MDR bacterial strains. The results showed a synergistic effect of the NP–antibiotic complex. These results indicated that the efficiency of NPs could still be further improved by combining them with antibiotics against both Gram-positive and Gram-negative clinical isolates. The results are reported in Figure 9 and Table 6.

### 3.5. Evaluation of Kinetic Action of NPs

**Figure 10:**
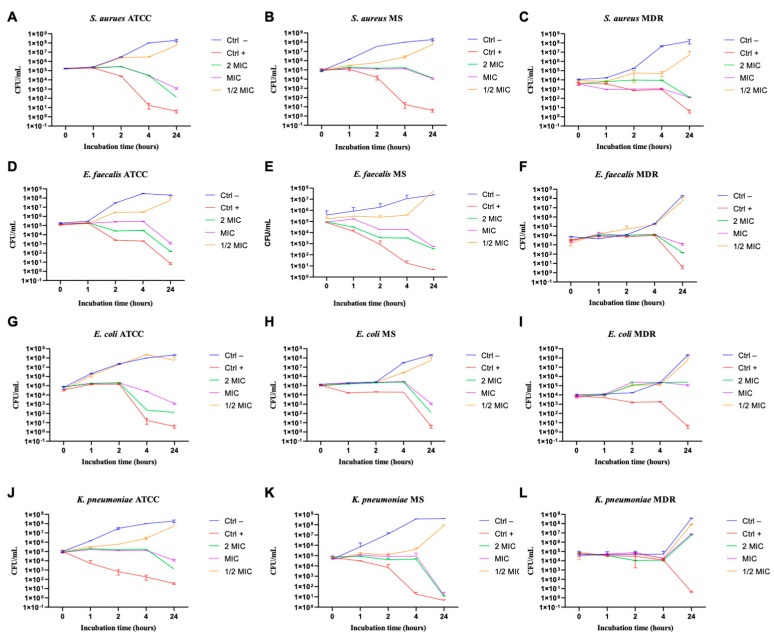
Bactericidal and bacteriostatic effect of AgPtNPs against different bacteria. (**A**–**C**) indicate the analysis against all *S. aureus* strains, (**D**–**F**) against *E. faecalis*, (**G**–**I**) against *E. coli*, and (**J**–**L**) against *K. pneumoniae*. Data are means of three independent experiments. CTR+ indicates bacteria treated with antibiotic, while CTR− represents not-treated bacteria. Data are relative to Ab- and represent the mean ± standard deviation (SD).

To understand the NPs’ kinetic action against the tested bacteria a time-killing assay was performed. The data obtained showed exponential bacterial growth over time without treatment (CTRL–) and with NPs ½ × MIC value. Exposure to 2xMIC and MIC value maintained the bacterial load at constant over time, suggesting a bacteriostatic action. A significant decrease in the CFU number of ATCC, MS, and MDR bacterial pathogens was observed after 24 h of incubation with AgPtNPs. The only exception was the MDR *E. coli* and *K. pneumoniae* strains, which showed comparatively less reduction in CFU. The overall results showed a partial bactericidal effect. On the contrary, NPs showed a bacteriostatic effect against *E. coli* (Figure 10G–I) and *K. pneumoniae* (Figure 10J,L).

### 3.6. Evaluation of Cell Viability against NPs Treatment

**Figure 11:**
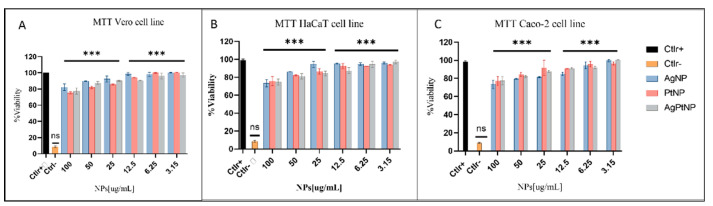
MTT assay on different cells lines. (**A**) Vero cells (**B**) HaCaT cells and (**C**) Caco-2 cells. Not treated cells were used as positive control (Ctlr+), while DMSO was used as negative control (Ctlr−). Data are means of three independent experiments ***: *p* < 0.001; ns: not significant, relative to Ctlr+.

To assess toxicity of NPs, we performed a cell viability assay on Vero cell line, HaCaT cell line and Caco-2 cell line after treatment with bimetallic NPs (AgPtNPs) and monometallic NPs (AgNPs and PtNPs) in a concentration range between 100 to 3.15 µg/mL. DMSO was used as negative control and non-treated cells as a positive control of viability. As reported in Figure 11, both bimetallic, and then monometallic NPs, did not affect cell viability until a higher test concentration of 100 µg/mL with an 80% survival rate in all tested cell lines (Figure 11). The results showed a non-toxic effect at the highest concentration of the NPs. The result was quite promising, compared to previous bimetallic NPs from plant extracts [13,31].

## 4. Discussion

Currently, multi-drug resistance and the side effects correlated with use of conventional drugs, require a search for new potential sources to fight different bacterial pathogens. In particular, researchers are focusing their attention on increasing hospital- and community-acquired infections due to multidrug-resistant (MDR) bacteria, against which current antibiotic therapies are not effective. Actually, nanoparticles and, in particular, silver nanoparticles (AgNPs), are considered a good alternative to antibiotics with a high potential to fight the problem of the emergence of bacterial multidrug resistance [32]. Silver has been used as an antiseptic and antimicrobial against Gram-positive and Gram-negative bacteria, due to its low cytotoxicity [33]. For the synthesis of NPs, only a few medicinal plants are used, namely, *Callicarpa maingayi*, *Cissus quadrangularis*, *Tribulus terrestris*, *Centella asiatica*, *Murraya koenigii*, *Alternanthera sessilis*, and *Artemisia nilagirica* [34]. Mainly, the reported plants are used to synthesize AgNPs, AuNPs, CuNPs, and other NPs. Only a few studies have shown the synthesis of bimetallic NPs (Au-Pd) from *Cacumen platycladi* leaf extract [35]. In this study, to synthesize bimetallic NPs, we used *O. basilicum* as a reducing agent, and the consortium of noble metals, namely silver and platinum. *O. basilicum* has exceptional medicinal benefits. Saba et al. [8] reported the synthesis of monometallic NPs in the presence of *O. basilicum*. During the synthesis of the NPs, the color change of the reaction, after adding the metal stock solution, indicated the reduction of metal ions during the reaction. The intensity of the color depended on time. As time increased, the reaction color became more intense. A preliminary analytical evaluation was performed in order to gain information about the chemical–physical properties of the nanoparticles. The UV-Vis spectroscopy data of our NPs showed the synthesis of bimetallic NPs, represented by peak formation at 320 nm. The previous studies also showed a peak formation ranging from 300 to 800 nm [36]. Overall, the shape of the AgPtNPs was found to be spherical with an average size of 49 nm, very similar to other previous studies [13]. The potential stability of NPs depends on the magnitude of zeta potential. Zeta values in the range of −30 to +30 are considered the most stable. In this study, the zeta value was −16, so the particles were considered stable. It has also been reported that small size and negative zeta potential provide good antimicrobial activity [13,37].

The phytoconstituents of the medicinal plant serve as a better precursor in reducing metal ions. The FTIR analysis of AgPtNPs from *O. basilicum* also showed a functional group related to amino acids, flavonoids, and proteins in the plant extract. A strong peak was observed at 1079 cm^−1^. The peak observed at 3353 represented N-H stretching. Likewise, the peaks obtained at 1023, 1265, 1384, and 2928 cm^−1^ were associated with cyclohexane ring vibration, C-H in-plane bend, NH_3_ ion, and the C-H stretching band in malignant and normal tissue stretching C-H, CH_2_ lipids, CH_2_ and aliphatic 2° amine, >N-H stretch, respectively [8,38]. Along with the stability, the morphology of NPs also plays an important role. In this study, we found that the shape of AgPtNPs was spherical with an inner core of silver and an outer coat of platinum. TEM analysis of the monometallic NPs was performed to confirm the difference. The AgNPs exhibited a small spherical structure with an average size of less than 20 nm, meanwhile the PtNPs showed a hollow morphology with a size of around 30 nm.

In the present study we evaluated the synthesized bimetallic and monometallic NPs against different clinical pathogens to assess their antimicrobial potential. We assessed the cytotoxicity of different cellular models by MTT assay. They were not toxic at all tested concentrations, maintaining cell proliferation in a rate of 80%. The results showed a non-toxic effect at the highest concentration of the NPs. The result was quite promising, compared to previous bimetallic NPs from plant extracts [13,31]. We tested the antibacterial potential of the NPs using different microbiological assays. The Kirby–Bauer method showed a significant inhibitory effect on the growth of the tested bacteria and the AgPtNPs showed a zone of inhibition for Gram-positive and Gram-negative bacterial strains in a range from 9–25 mm. While the AgNPs showed an inhibition zone of 9–10 mm, the PtNPs showed inhibition zones in a range of 6–9 mm. In comparison with antibiotics used as positive control, Vancomycin for Gram-positive and Ampicillin for Gram negative, we were able to assess that the bimetallic composition was more effective, in respect to monometallic alone. These results, together with the MIC assay results, indicated that the AgPtNPs were strongly active till the least concentration of NPs (3.12–1.56 µg/mL), while the monometallic AgNPs showed MIC in the range of 25–3.15 µg/mL, and for PtNPs it was 12.5–3.12 µg/mL. The antimicrobial effect of our bimetallic NPs appeared more potent than previously reported [2,24,31]. Our results were consistent with literature data, although differences were found due to growth condition variations and/or bacterial strains investigated. We also performed a checkerboard assay to check the efficiency of the bimetallic nanoparticles on a combination of antibiotics. The results supported the synergistic behavior of the bimetallic NPs in the presence of antibiotics.

The mechanism of action of AgNPs has already been well explained, but for the AgPtNPs it is not yet known. However, a study by Vazquez-Munoz [39] showed that AgNPs could alter the cell membrane, causing leakage, and, finally, bacterial death. On the other hand, another study demonstrated that Ti–PtNPs caused bacterial death due to leakage of cytosolic protein [40]. Based on this, we assume that AgPtNPs might firmly bind to the bacterial cell wall, which may generate oxidative stress. This could also lead to the development of superoxide [13]. The AgPtNPs might interact with nucleic acid and interrupt cellular transport synthesis. However, the pharmacokinetic implications of these NPs and their mechanism of action require more detailed investigation. Use of *O. basilicum*, due to its bioactive chemical constituents, such as polyphenols, flavonoids, glycosides, and alkaloids, limits our full understanding of their therapeutic effectiveness [41]. Finally, considering the growing global interest in “green production” to address cogent problems of antimicrobial resistance, functionalizing green AgPtNps derived from these *O. basilicum* pharmacologically important plants is an option in the preparation of medicinal plants in combination with emergent nano therapy methods [42] Indeed, it is expected that the enhanced biocatalytic activity of AgPtNPs, which contain in situ generated capping agents, synthesized from the leaf of *O. basilicum*, in combination, may have the capacity to serve as antimicrobial agents.

## 5. Conclusions

In this study, we synthesized AgPtNPs using *O. basilicum* to reduce silver and platinum salts. In this method, we observed improved stability of the AgPtNPs by analytical tools, such as DLS zeta potential, FTIR, and TEM. The average size of the NPs was found to be ~59nm, with a negative zeta potential of −16mV. Due to the smaller size of the NPs and the negative zeta potential, the NPs had good antimicrobial activity against all tested ATTC, MS, and MDR bacterial strains. Not only tested organisms showed susceptibility to AgPtNPs alone, but there were also a synergistic effect in the presence of antibiotics. The FTIR study showed that the presence of different biochemicals explains the stability and efficient capping on the surface of the NPs. This could be one reason for the high survival rate of more than 80% in different cell lines while treating with the NPs. A future study on the conjugation of antibiotics on the surface of NPs could be enhanced due to the stability of these NPs. Based on their antimicrobial properties, green-synthesized bimetallic NPs from a natural source, such as *O. basilicum* leaf extract, could be used as an innovative antibacterial strategy to counteract the cogent problem of antimicrobial resistance.

## Figures and Tables

**Table 1:** Antibiotic resistance profiles of ATCC strains used in the study (S: susceptibility; R: resistant bacterial strain).

Antibiotic Resistance Profile of the ATCC Bacterial Strains		
Antibiotics	MIC (mg/L)	Interpretation
*S. aureus* ATCC 6538		
Fusidic acid	≤0.5	S
Daptomycin	0.25	S
Erythromycin	≤0.25	S
Fosfomycin	≤8	S
Gentamicin	≤0.5	S
Linezolid	2	S
Levofloxacin	≤0.12	S
Oxacillin	≤0.25	S
Teicoplanin	≤0.5	S
Tetracycline	≤1	S
Tigecycline	≤0.12	S
Trimethoprim/sulfamethoxazole	≤10	S
Vancomycin	0.5	S
Penicillin	≤0.03	S
Rifampicin	≤0.03	S
*E. faecalis* ATCC 29212		
Ampicillin	2	S
Gentamicin/syn	500	S
Imipenem	1	S
Linezolid	2	S
Teicoplanin	0.5	S
Tigecycline	0.12	S
Vancomycin	2	S
Cefuroxime	64	R
*E. coli* ATCC 11229		
Amikacin	2	S
Amoxicillin/clavulanate	2	S
Ampicillin	8	S
Cefepime	1	S
Cefotaxime	1	S
Ceftazidime	1	S
Cefuroxime	4	S
Ciprofloxacin	0.25	S
Ertapenem	0.5	S
Fosfomycin	16	S
Gentamicin	1	S
Imipenem	0.25	S
Levofloxacin	0.5	S
Meropenem	0.25	S
Piperacillin	8	S
Piperacillin/tazobactam	4	S
Tobramycin	1	S
Trimethoprim/sulfamethoxazole	20	S
Tigecycline	0.5	S
*K. pneumoniae* ATCC 10031		
Ciprofloxacin	0.25	S
Fosfomycin	16	S
Ampicillin	8	S
Gentamicin	1	S
Trimethoprim/sulfamethoxazole	20	S
Amikacin	2	S
Amoxicillin/clavulanate	2	S
Cefepime	1	S
Cefotaxime	1	S
Cefotaxime	1	S
Ertapenem	0.5	S
Imipenem	0.5	S
Meropenem	0.25	S
Piperacillin/tazobactam	4	S
Colistin	0.5	S

**Table 2:** Antibiotic resistance profiles of the clinical isolates used in the study [20].

Antibiotic Resistance Profile of the Clinical Isolated Bacteria		
Antibiotics	MIC (mg/L)	Interpretation
*S. aureus* MS		
Fusidic acid	0.5	S
Daptomycin	0.25	S
Erythromycin	0.25	S
Fosfomycin	8	S
Gentamicin	0.5	S
Linezolid	2	S
Levofloxacin	0.12	S
Oxacillin	0.25	S
Teicoplanin	0.5	S
Tetracycline	1	S
Tigecycline	0.12	S
Trimethoprim/sulfamethoxazole	10	S
Vancomycin	0.5	S
Penicillin	0.03	S
*S. aureus* MDR		
Fusidic acid	0.5	S
Daptomycin	0.25	S
Erythromycin	4	R
Fosfomycin	64	R
Gentamicin	8	R
Linezolid	2	S
Levofloxacin	8	R
Oxacillin	2	R
Teicoplanin	0.5	S
Tetracycline	1	S
Tigecycline	0.12	S
Trimethoprim/sulfamethoxazole	20	S
Vancomycin	0.5	S
Penicillin	0.25	R
Rifampicin	2	R
*E. faecalis* MS		
Ampicillin	2	S
Gentamicin/syn	500	S
Imipenem	2	S
Linezolid	2	S
Teicoplanin	0.5	S
Tigecycline	0.25	S
Vancomycin	2	S
Cefuroxime	64	R
*E. faecalis* MDR		
Ampicillin	8	R
Gentamicin/syn	500	R
Imipenem	8	R
Linezolid	2	S
Teicoplanin	0.5	S
Tigecycline	0.25	R
Vancomycin	2	S
*E. coli* MS		
Amikacin	2	S
Amoxicillin/clavulanate	2	S
Ampicillin	8	S
Cefepime	1	S
Cefotaxime	1	S
Ceftazidime	1	S
Cefuroxime	4	S
Ciprofloxacin	0.25	S
Ertapenem	0.5	S
Fosfomycin	16	S
Gentamicin	1	S
Imipenem	0.25	S
Levofloxacin	0.5	S
Meropenem	0.25	S
Piperacillin	8	S
Piperacillin/tazobactam	4	S
Tobramycin	1	S
Trimethoprim/sulfamethoxazole	20	S
Tigecycline	0.5	S
*E. coli* MDR		
Amikacin	16	R
Amoxicillin/clavulanate	32/2	R
Ampicillin	8	R
Cefepime	8	R
Cefotaxime	4	R
Ceftazidime	8	R
Cefuroxime	8	R
Ciprofloxacin	1	R
Ertapenem	0.25	R
Fosfomycin	16	R
Gentamicin	4	R
Imipenem	0.25	R
Levofloxacin	2	R
Meropenem	0.125	R
Piperacillin	16	R
Piperacillin/tazobactam	16/4	R
Tobramycin	4	R
Trimethoprim/sulfamethoxazole	1/19	R
Tigecycline	1	R
*K. pneumoniae* MS		
Ciprofloxacin	0.25	S
Fosfomycin	16	S
Ampicillin	1	S
Gentamicin	20	S
Trimethoprim/sulfamethoxazole	2	S
Amikacin	8	S
Amoxicillin/clavulanate	2	S
Cefepime	1	S
Cefotaxime	1	S
Cefotaxime	1	S
Ertapenem	0.5	S
Imipenem	0.25	S
Meropenem	0.25	S
Piperacillin/tazobactam	4	S
Colistin	0.5	S
*K. pneumoniae* MDR		
Ciprofloxacin	4	R
Fosfomycin	64	S
Ampicillin	1	S
Gentamicin	320	R
Trimethoprim/sulfamethoxazole	64	R
Amikacin	32	R
Amoxicillin/clavulanate	8	R
Cefepime	64	R
Cefotaxime	64	R
Cefotaxime	64	R
Ertapenem	8	R
Imipenem	16	R
Meropenem	16	R
Piperacillin/tazobactam	128	R
Colistin	0.5	S

**Table 3:** Inhibition zone (mm) of AgPtNPs against ATCC, MS and MDR bacterial strains. (±SD 3 replicates tested). Positive Control is Vancomycin for Gram-positive and Ampicillin for Gram-negative.

Bacterial Strain	Zone of Inhibition (mm)			
	AgPtNPs	AgNPs Alone	PtNPs Alone	Control Positive
*S. aureus ATCC*	14.3 ± 1.374	9.3 ± 0.608	6.2 ± 0.264	30.76 ± 0.737
*S. aureus MS*	18.56 ± 1.040	9.03 ± 0.057	7.76 ± 0.251	31.3 ± 0.793
*S. aureus MDR*	21.43 ± 0.896	10.66 ± 0.577	9.43 ± 0.513	31.72 ± 2.44
*E. coli ATCC*	13.87 ± 1.205	8.33 ± 0.577	6.46 ± 0.503	37.76 ± 2.21
*E. coli MS*	9.633 ± 0.721	6.23 ± 0.321	6 ± 0	36.63 ± 1.35
*E. coli MDR*	14.9 ± 1.069	8.8 ± 0.435	6.83 ± 0.288	38.86 ± 1.001
*E. faecalis ATCC*	18.9 ± 0.950	9.56 ± 0.513	9.53 ± 0.50	30.73 ± 0.763
*E. faecalis MS*	21.033 ± 0.650	10.96 ± 0.950	9.2 ± 0.346	29.46 ± 0.602
*E. faecalis MDR*	23.16 ± 0.472	9.9 ± 0.854	9.8 ± 0.173	29.5 ± 1.539
*K. pneumoniae ATCC*	13.63 ± 0.550	9.63 ± 0.550	6.66 ± 0.577	37.2 ± 2.264
*K. pneumoniae MS*	18.4 ± 0.781	7.3 ± 0.608	6.33 ± 0.577	38.53 ± 1.266
*K. pneumoniae MDR*	19.86 ± 0.115	9.6 ± 0.529	8.83 ± 0.288	38.46 ± 1.3012

**Table 4:** Minimum inhibitory concentration of bimetallic and monometallic NPs against all Gram-positive bacteria.

Bacterial Strain	MIC Value (µg/mL)		
	AgPtNPs	AgNPs	PtNPs
*S. aureus* *ATCC*	3.12	6.25	12.5
*S. aureus MS*	1.56	3.12	3.12
*S. aureus MDR*	1.56	6.25	3.12
*E. faecalis* *ATCC*	3.12	3.12	6.25
*E. faecalis MS*	3.12	6.25	6.25
*E. faecalis MDR*	3.12	6.25	12.5

**Table 5:** Minimum inhibitory concentration of bimetallic and monometallic NPs against all Gram-negative bacterial strains.

Bacterial Strain	MIC Value (µg/mL)		
	AgPtNPs	AgNPs	PtNPs
*E. coli ATCC*	1.56	25	25
*E. coli MS*	1.56	6.25	6.25
*E. coli MDR*	3.25	6.25	12.5
*K. pneumoniae ATCC*	3.25	12.5	12.5
*K. pneumoniae MS*	1.56	12.5	6.25
*K. pneumoniae MDR*	1.56	6.25	6.25

**Table 6:** Interpretation of checkerboard assay.

Test Organism	FIC Index	Effect
*S. aureus* *ATCC*	0	Synergistic effect
*S. aureus MS*	0.46	Synergistic effect
*S. aureus MDR*	0.5	Synergistic effect
*E. coli ATCC*	0.326	Synergistic effect
*E. coli MS*	0.45	Synergistic effect
*E. coli MDR*	0.376	Synergistic effect
*E. faecalis* *ATCC*	0.46	Synergistic effect
*E. faecalis MS*	0.66	Additive effect
*E. faecalis MDR*	0.5	Additive effect
*K. pneumoniae ATCC*	0.31	Synergistic effect
*K. pneumoniae MS*	0.31	Synergistic effect
*K. pneumoniae MDR*	0.75	Additive effect

## Data Availability

The data presented in this study are available herein.
